# Transient Receptor Potential vanilloid 4 ion channel in C-fibres is involved in mechanonociception of the normal and inflamed joint

**DOI:** 10.1038/s41598-019-47342-x

**Published:** 2019-07-29

**Authors:** Frank Richter, Gisela Segond von Banchet, Hans-Georg Schaible

**Affiliations:** Institute of Physiology 1, Jena University Hospital, Friedrich Schiller University Jena, Jena, Germany

**Keywords:** Excitability, Sensory processing

## Abstract

The Transient Receptor Potential vanilloid 4 ion channel (TRPV4) is an important sensor for osmotic and mechanical stimuli in the musculoskeletal system, and it is also involved in processes of nociception. In this study we investigated the putative role of TRPV4 ion channels in joint pain. In anesthetized rats we recorded from mechanosensitive nociceptive A∂- and C-fibres supplying the medial aspect of the knee joint. The intraarticular injection of the TRPV4 antagonist RN-1734 into the knee joint reduced the responses of C-fibres of the normal joint to noxious mechanical stimulation and the responses of the sensitized C-fibres of the acutely inflamed joint to innocuous and noxious mechanical stimulation. The responses of nociceptive A∂-fibres were not significantly altered by RN-1734. The intraarticular application of the TRPV4 agonists 4αPDD, GSK 1016790 A, and RN-1747 did not consistently alter the responses of A∂- and C-fibres to mechanical stimulation of the joint nor did they induce ongoing activity. We conclude that TRPV4 ion channels are involved in the responses of C-fibres to noxious mechanical stimulation of the normal joint, and in the enhanced sensitivity of C-fibres to mechanical stimulation of the joint during inflammation of the joint.

## Introduction

Mechanical stimuli play a major role in joint pain. In a normal joint pain is elicited by movements which exceed the working range of the joint. When the joint is inflamed, pain is even elicited by movements in the working range of the joint^1–3^. An important basis of this pattern of joint pain are the response properties of joint nociceptors. Noxious mechanical stimuli applied to the joint (movements exceeding the working range and intense pressure applied to the joint) are encoded by Aδ- and C-fibres which are selectively activated by noxious mechanical stimuli, and by Aδ- and C-fibres which are only weakly activated by innocuous stimuli (movements within the working range, light to moderate local pressure) but respond strongly to noxious mechanical stimuli^1,2^. During inflammation, nociceptive Aδ- and C-fibres are sensitized. In the sensitized state, the nociceptive fibres show reduced thresholds for activation and increased responses to innocuous and noxious stimuli, thus explaining why mechanical stimuli applied to the inflamed joint are painful^1,2^.

It is unclear which ion channels in nociceptive neurons are involved in the transduction of mechanical stimuli applied to the joint. A candidate is the Transient Receptor Potential (TRP) vanilloid 4 ion channel (TRPV4). This ion channel, discovered in 2000^4,5^, is a polymodally activated Ca^2+^-permeable non-selective cation channel that is involved in the transduction of osmotic and mechanical stimuli^6^. Importantly, TRPV4 is also involved in nociception^7^. The channel is expressed in sensory ganglia and free nerve endings in the skin^7^. In behavioural experiments TRPV4 mediated nocifensive behaviours to increases or decreases of osmolarity^8,9^ and plays a crucial role in mechanical hyperalgesia following inflammatory mediators^10^. TRPV4 knockout mice showed less inflammatory and thermal hyperalgesia induced by capsaicin or carrageenan^11^, impaired sensitivity to acid, an increase in mechanical nociceptive threshold, altered thermal selection behaviours but normal responses to heat and low-threshold mechanical stimuli^12,13^. TRPV4 may promote the release of SP and CGRP from sensory neurons^14^. A recent study showed that antagonists at the TRPV4 ion channel partially reversed the reduction of grip strength in a model of osteoarthritis^15^ suggesting that TRPV4 may also play a role in joint pain.

In the present study we explored the role of TRPV4 in the nociception of the joint *in vivo*. We recorded from nociceptive sensory fibres of the knee joint in anesthetized rats. This is a well-established approach to investigate the role of mediators in mechanonociception^1,16–18^ because it allows to repeatedly stimulate joint nociceptors by noxious mechanical stimuli and to explore how the local application of mediators to the joint alters the responsiveness of the nociceptive nerve fibres to mechanical stimuli. Therefore, single fibre recordings bridge the gap between cellular/molecular mechanisms and behavioural studies which can only measure pain thresholds^1,16–18^. For study we selected single nociceptive A∂- and C-fibres with a receptive field in the medial aspect of the exposed knee joint. We asked whether the responses to innocuous and noxious rotations are altered by the application of TRPV4 agonists and a TRPV4 antagonist^19^ into the knee joint cavity. Recordings were performed in rats with healthy knee joints and in rats in which the knee joint was acutely inflamed.

## Results

We recorded from single Aδ- and C-fibres of 73 anesthetized rats (usually one fibre per rat). Each sensory fibre had a receptive field in the medial aspect of the knee joint and responded weakly to innocuous but strongly to noxious rotation of the joint. None of the recorded fibres showed ongoing activity. The first sections will describe the effects of the TRPV4 antagonist on the responses to mechanical stimulation.

### Effect of the TRPV4 receptor antagonist RN-1734 on the responses of joint afferents to mechanical stimuli applied to the normal knee joint

**Figure 1:**
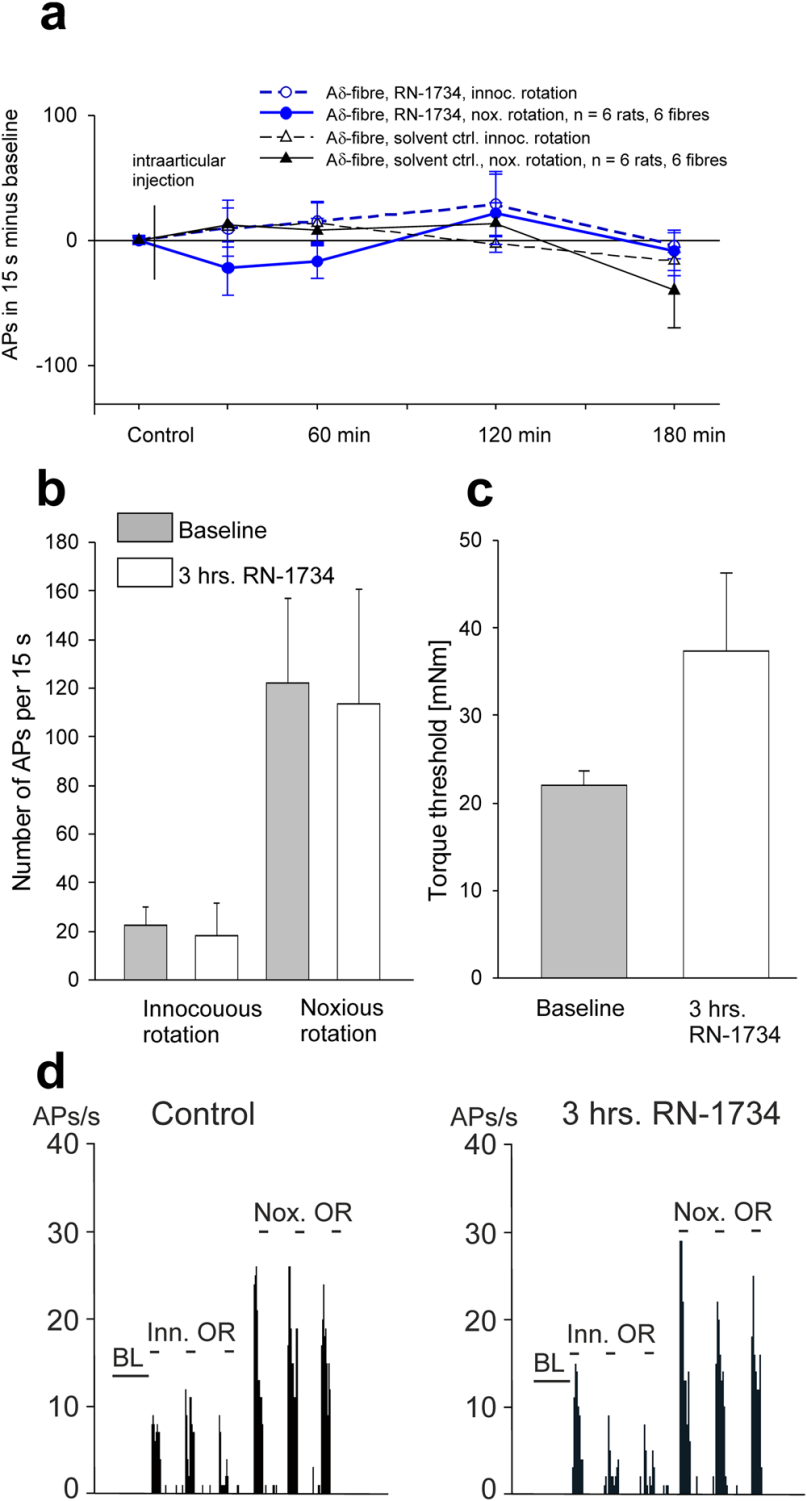
Influence of the TRPV4 antagonist RN-1734 (500 µM) on the responses of A∂-fibres of the normal joint to innocuous and noxious rotation. (**a**) Time course of the change of the responses to innocuous and noxious rotation after intraarticular injection of either the solvent or RN-1734. In all tested fibres the baseline value before the intraarticular injection (control) was set zero. Baseline values: innocuous rotation solvent group 26 ± 8 APs/15 s, innocuous rotation RN-1734 group 23 ± 8 APs/15 s, noxious rotation solvent group 102 ± 45 APs/15 s, noxious rotation RN-1734 group 122 ± 35 APs/15 s. (**b**) Responses of A∂-fibres to innocuous and noxious rotation of the normal joint before and at 3 hours after intraarticular application of RN-1734. (**c**) Torque threshold for the elicitation of action potentials before and at 3 hours after RN-1734. (**d**) Specimen of an A∂-fibre. Peristimulus histograms show the responses to innocuous and noxious outward rotation in the control period and at 3 hours after RN-1734. Ctrl: control, Inn(oc): innocuous, nox: noxious, AP: action potential, OR: outward rotation, BL: baseline.

Figure 1a shows the average changes of the responses of 12 Aδ-fibres from 12 knee joints to movement stimuli of the normal knee joint at different time points. In 6 knee joints (6 fibres) only the solvent was injected into the knee joint, in another 6 knee joints (6 fibres) the antagonist RN-1734 was injected. The initial baseline responses (control) before the intraarticular injection of the solvent or the antagonist were set to zero, and the different lines show the changes of the responses compared to the baseline value. Neither the solvent nor RN-1734 (500 µM) significantly altered the responses to innocuous and noxious rotation of the joint (Fig. 1a). Figure 1b displays the number of action potentials elicited by innocuous and noxious rotation at baseline and at 3 hours after the application of the antagonist. On average, the A∂-fibres showed a weak response to innocuous rotation and a much stronger response to noxious rotation, and these responses were not altered by RN-1734. We also determined the torque, necessary for the elicitation of a response to rotation. After application of RN-1734 the mechanical torque thresholds increased non-significantly from 22.0 ± 1.6 to 37.4 ± 8.9 mNm (Fig. 1c). The peristimulus time histograms in the specimen in Fig. 1d display the responses of a typical A∂-fibre before and at 3 hours after application of RN-1734.

**Figure 2:**
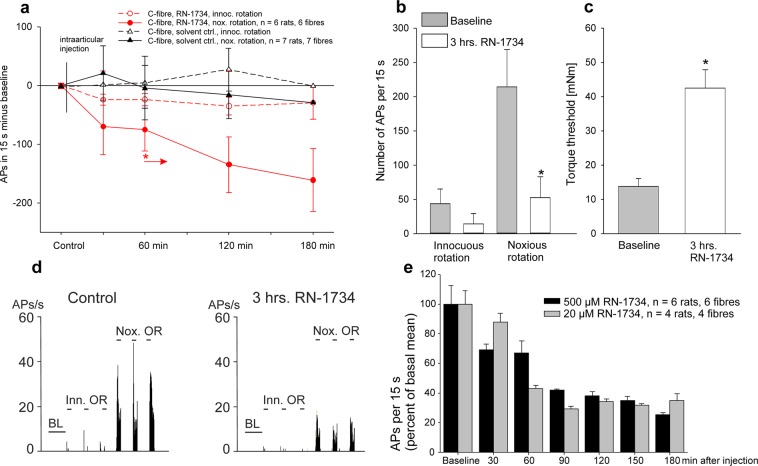
Influence of the TRPV4 antagonist RN-1734 (500 µM and 20 µM) on the responses of C-fibres of the normal joint to innocuous and noxious rotation. (**a**) Time course of the change of the responses to innocuous and noxious rotation after intraarticular injection of either the solvent or RN-1734. In all tested fibres the baseline value before the intraarticular injection (control) was set zero. Baseline values: innocuous rotation solvent group 79 ± 28 APs/15 s, innocuous rotation RN-1734 group 44 ± 21 APs/15 s, noxious rotation solvent group 229 ± 46 APs/15 s, noxious rotation RN-1734 group 214 ± 55 APs/15 s. (**b**) Responses of C-fibres to innocuous and noxious rotation of the normal joint before and at 3 hours after intraarticular application of RN-1734. (**c**) Torque threshold for the elicitation of action potentials before and at 3 hours after RN-1734. (**d**) Specimen of a C-fibre. Peristimulus histograms show the responses to innocuous and noxious outward rotation in the control period and at 3 hours after RN-1734. (**e**) Comparison of the effect of 500 µM RN-1734 (same data as in a) and of 20 µM RN-1734 on the responses to noxious rotation of the knee joint. The columns show the reduction from baseline at defined time points. *In (**a**,**b**) p < 0.0313, Wilcoxon signed rank test, *in (**c**) p < 0.0047, paired t-test.

With the same protocol we tested the effect of RN-1734 (500 µM) on the responses of 6 C-fibres from 6 joints, and the solvent was tested in another 7 C-fibres from 7 normal knee joints (Fig. 2). The responses to innocuous and noxious rotations were not significantly altered after application of the solvent into the joint cavity. After the intraarticular application of RN-1734 the responses to innocuous rotation were not altered either but the responses to noxious rotation showed a consistent reduction (Fig. 2a, from the asterisk onwards the responses were significantly reduced compared to the baseline responses; p = 0.0313, Wilcoxon signed rank test). Figure 2b displays the average number of evoked action potentials per stimulus before and at 3 hours after the application of RN-1734, with a significant difference for noxious rotation only (p < 0.0313, Wilcoxon signed rank test). The torque, necessary for the elicitation of a response to rotation, significantly increased after RN-1734 from 13.8 ± 2.2 to 42.4 ± 5.5 mNm (Fig. 2c, p = 0.0047, paired t-test). The specimen of a single fibre in Fig. 2d shows a strong reduction of the responses at 3 hours after RN-1734.

In a further group of four rats we applied the antagonist RN-1734 at a dose of 20 µM. After RN-1734 at 20 µM the responses of the C-fibres showed a gradual decrease of the numbers of action potentials similar as the responses of the C-fibres after application of the high dose of RN-1734 in the other rats (Fig. 2e). The low dose of 20 µM did not influence the number of action potentials in A∂-fibres (data not shown). Thus, in the normal joint, the TRPV4 antagonist RN-1734 significantly reduced the responses of C-fibres to noxious rotation but did not influence the responses of A∂-fibres.

### Effect of the TRPV4 receptor antagonist RN-1734 on the responses of joint afferents to mechanical stimuli applied to the acutely inflamed knee joint

In further experiments we tested whether the antagonist influenced the responses of A∂-and C-fibres of the acutely inflamed joint to innocuous and noxious rotation. Inflammation was induced 7–11 hours before the recordings and was therefore fully developed.

**Figure 3:**
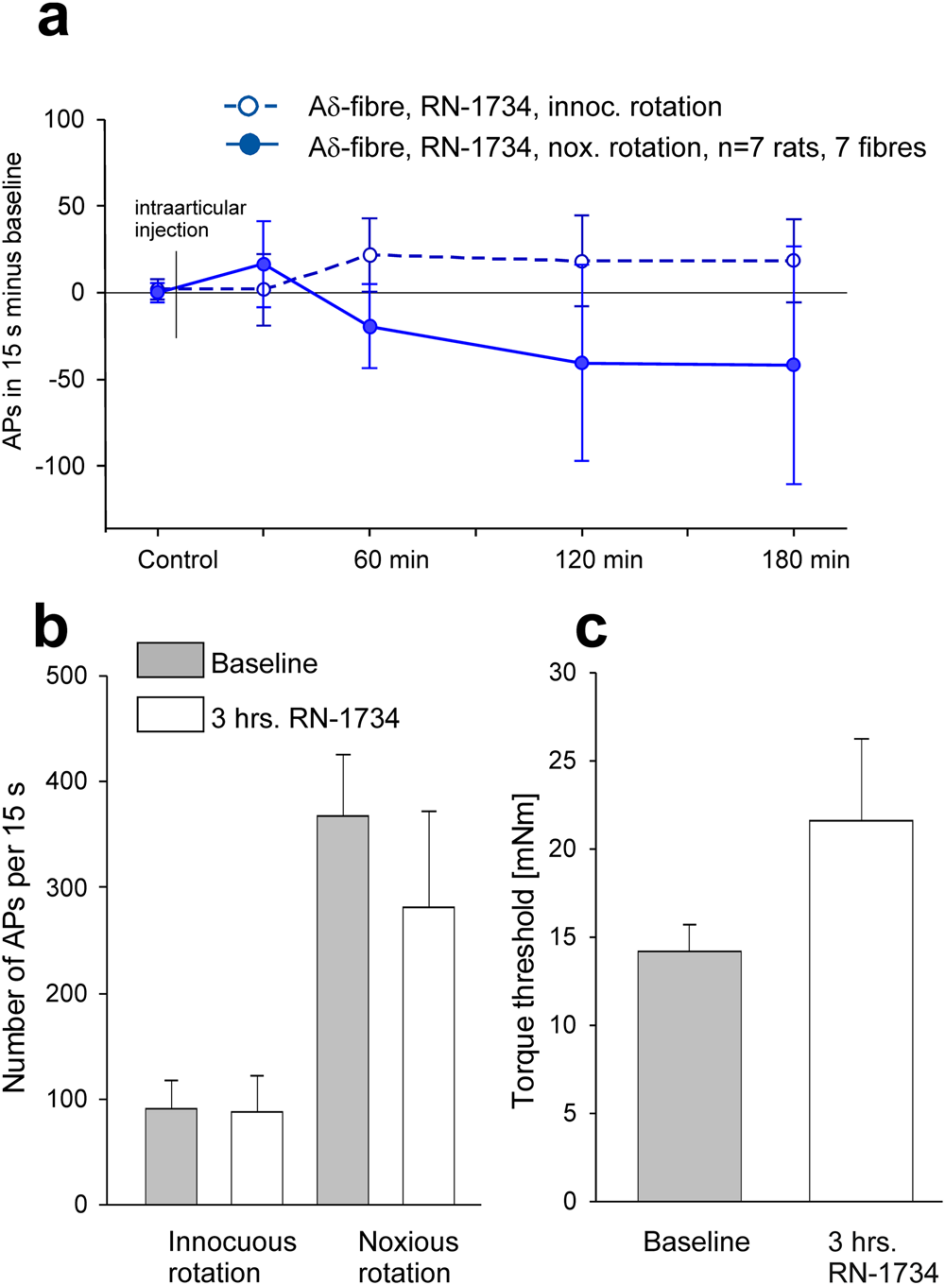
Influence of the TRPV4 antagonist RN-1734 (500 µM) on the responses of A∂-fibres of the acutely inflamed joint to innocuous and noxious rotation. (**a**) Time course of the change of the responses to innocuous and noxious rotation after intraarticular injection of RN-1734. In all tested fibres the baseline value before the intraarticular injection (control) was set zero. Baseline values: innocuous rotation RN-1734 group 90 ± 27 APs/15 s, noxious rotation RN-1734 group 367 ± 58 APs/15 s. (**b**) Responses of A∂-fibres to innocuous and noxious rotation of the inflamed joint before and at 3 hours after intraarticular application of RN-1734. (**c**) Torque threshold for the elicitation of action potentials before and at 3 hours after RN-1734. No significant differences.

The 7 A∂-fibres of 7 inflamed knee joints had on average stronger responses to innocuous and noxious rotation than the A∂-fibres of the normal joint (innocuous rotation normal joint 23 ± 8 APs/15 s, inflamed joint 90 ± 70 APs/15 s, noxious rotation normal joint 122 ± 35 APs/15 s, inflamed joint 367 ± 58 APs/15 s). However, on average the responses to mechanical stimuli were not significantly reduced by RN-1734 (Fig. 3a,b), and the threshold torque was not significantly influenced either (Fig. 3c).

**Figure 4:**
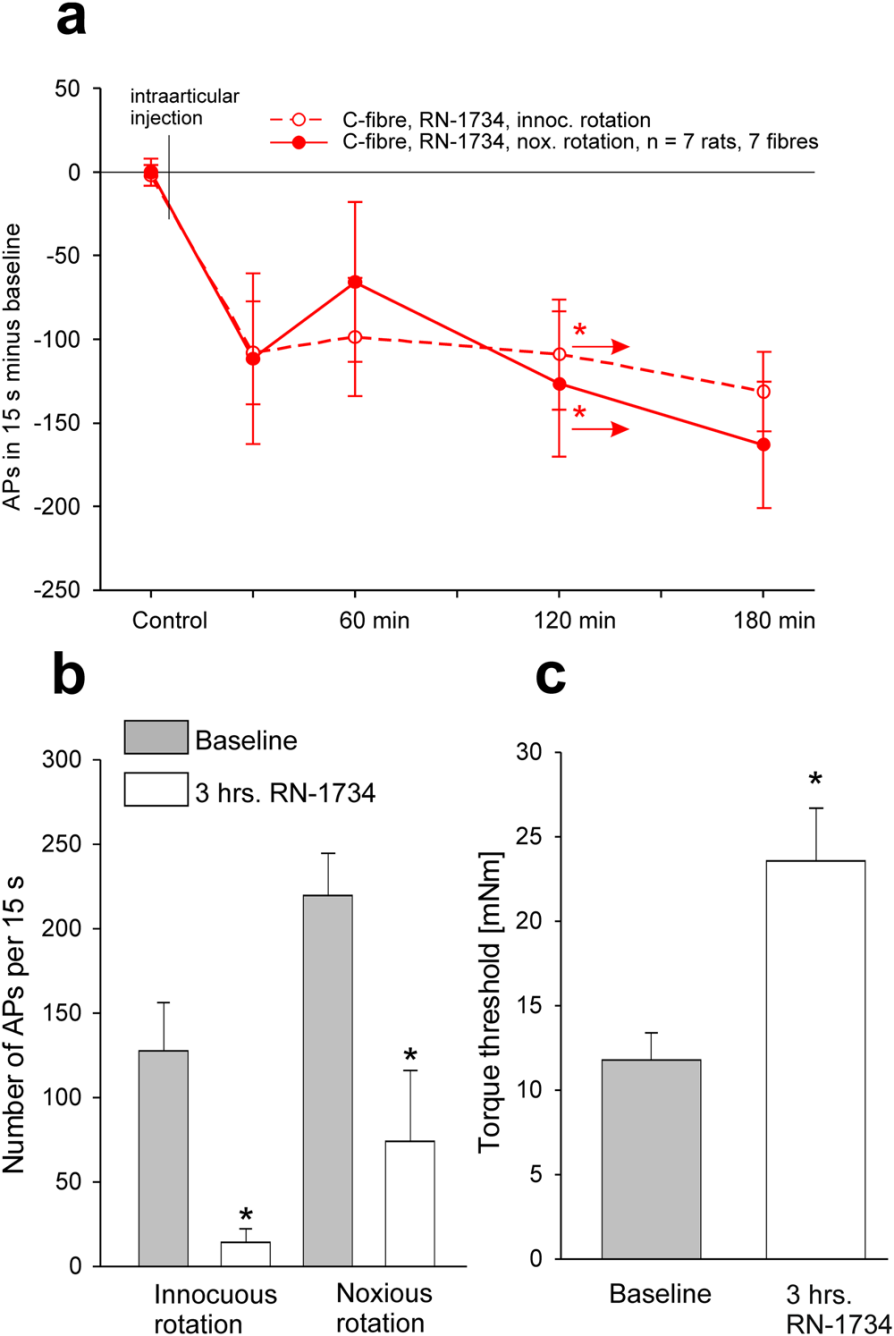
Influence of the TRPV4 antagonist RN-1734 (500 µM) on the responses of C-fibres of the acutely inflamed joint to innocuous and noxious rotation. (**a**) Time course of the change of the responses to innocuous and noxious rotation after intraarticular injection of RN-1734. In all tested fibres the baseline value before the intraarticular injection (control) was set zero. Baseline values: innocuous rotation RN-1734 group 128 ± 77 APs/15 s, noxious rotation RN-1734 group 219 ± 25 APs/15 s. (**b**) Responses of C-fibres to innocuous and noxious rotation of the inflamed joint before and at 3 hours after intraarticular application of RN-1734. (**c**) Torque threshold for the elicitation of action potentials before and at 3 hours after RN-1734. *In (**a**,**b**) p < 0.0469, Wilcoxon signed-rank test, *in (**c**) p < 0.0082, paired t-test.

In the 7 C-fibres of 7 inflamed knee joints in particular the baseline responses to innocuous rotation were enhanced compared to those in the C-fibres of normal joints (128 ± 75 APs/15 s versus 43 ± 21 APs/15 s) whereas the responses to noxious rotation had a similar magnitude as in the normal joint (compare Figs 2b and 4b). In the C-fibres of the inflamed knee joint both the responses to innocuous and noxious rotation were reduced by RN-1734 (p = 0.0469, Wilcoxon signed rank test, Fig. 4a,b), and the threshold torque for an elicitation of a response was enhanced by RN-1734 (p = 0.0082, paired t-test, Fig. 4c). Thus, in the inflamed knee joint the TRPV4 antagonist RN-1734 significantly reduced the responsiveness of the C-fibres to innocuous and noxious rotation but not the responsiveness of the A∂-fibres.

**Figure 5:**
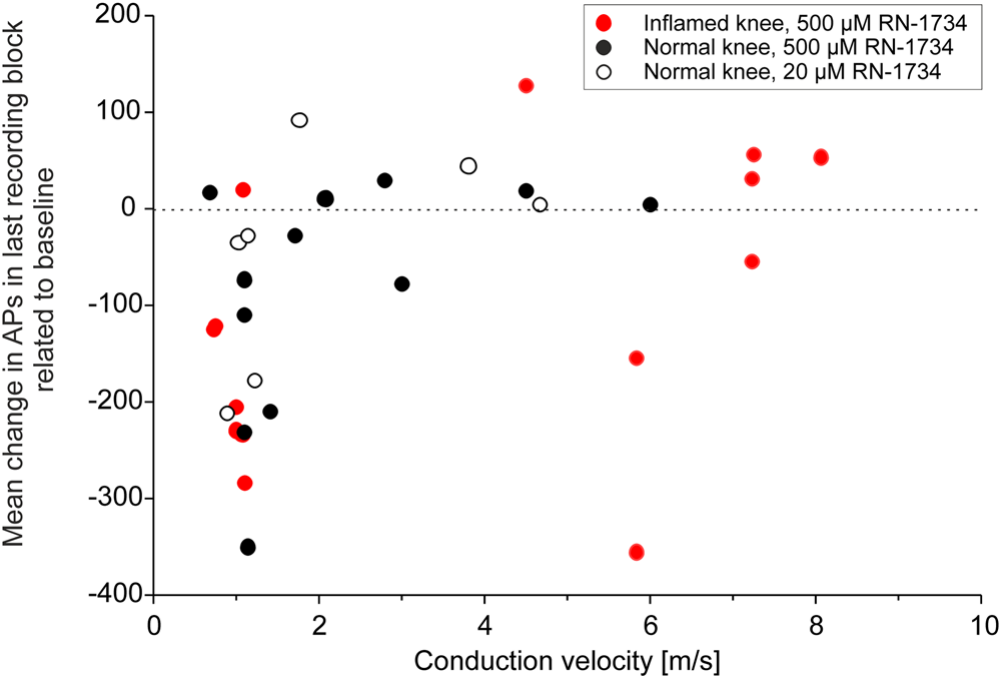
Influence of the TRPV4 antagonist RN-1734 on the responses of nerve fibres of the normal joint (black symbols) and the inflamed joint (red symbols) to noxious rotation. On the x-axis the conduction velocities of the fibres are shown in m/s, on the y-axis the normalised mean changes of the responses to noxious rotation in numbers of action potentials (APs) in the last recording block at three hours after intraarticular application of the antagonist (mean baseline AP numbers are subtracted from the actual numbers of APs).

Since the different effects of RN-1734 on the average responses of A∂-fibres and C-fibres suggested that mainly the C-fibres are influenced by RN-1734, we plotted in Fig. 5 all the fibres recorded from, showing their conduction velocity and the effect of RN-1734. It is evident that with few exceptions only C-fibres were influenced by the TRPV4 antagonist. In the normal joint (fibres with black dots and circles) a decrease of the responses after RN-1734 was mainly observed in fibres with conduction velocities < 2 m/s, i.e. C-fibres. In the inflamed joint, C-fibres and two fibres with a conduction velocity of about 6 m/s showed a reduction of the responses.

### Effects of TRPV4 receptor agonists on the responses of joint afferents to mechanical stimuli applied to the normal knee joint

We explored whether TRPV4 agonists influenced the responses of A∂- and C-fibres of the normal joint to innocuous and noxious stimulation. We chose the agonists 4αPDD, GSK 1016790 A, and RN-1747 which were used in different studies as specific TRPV4 agonists^19^.

We monitored action potentials throughout the experiments and therefore we were able to see the generation of action potentials evoked by the application of the compounds themselves. In the first few minutes after intraarticular injections of either buffer or a compound some irregular action potentials could occur in some experiments but no consistent pattern was observed. However, none of the agonists did evoke ongoing discharges between the stimuli throughout the recording protocol, in the absence of mechanical stimulation. Within the next 2–3 hours we then explored whether the responses of the fibres to mechanical stimulation are altered.

**Figure 6:**
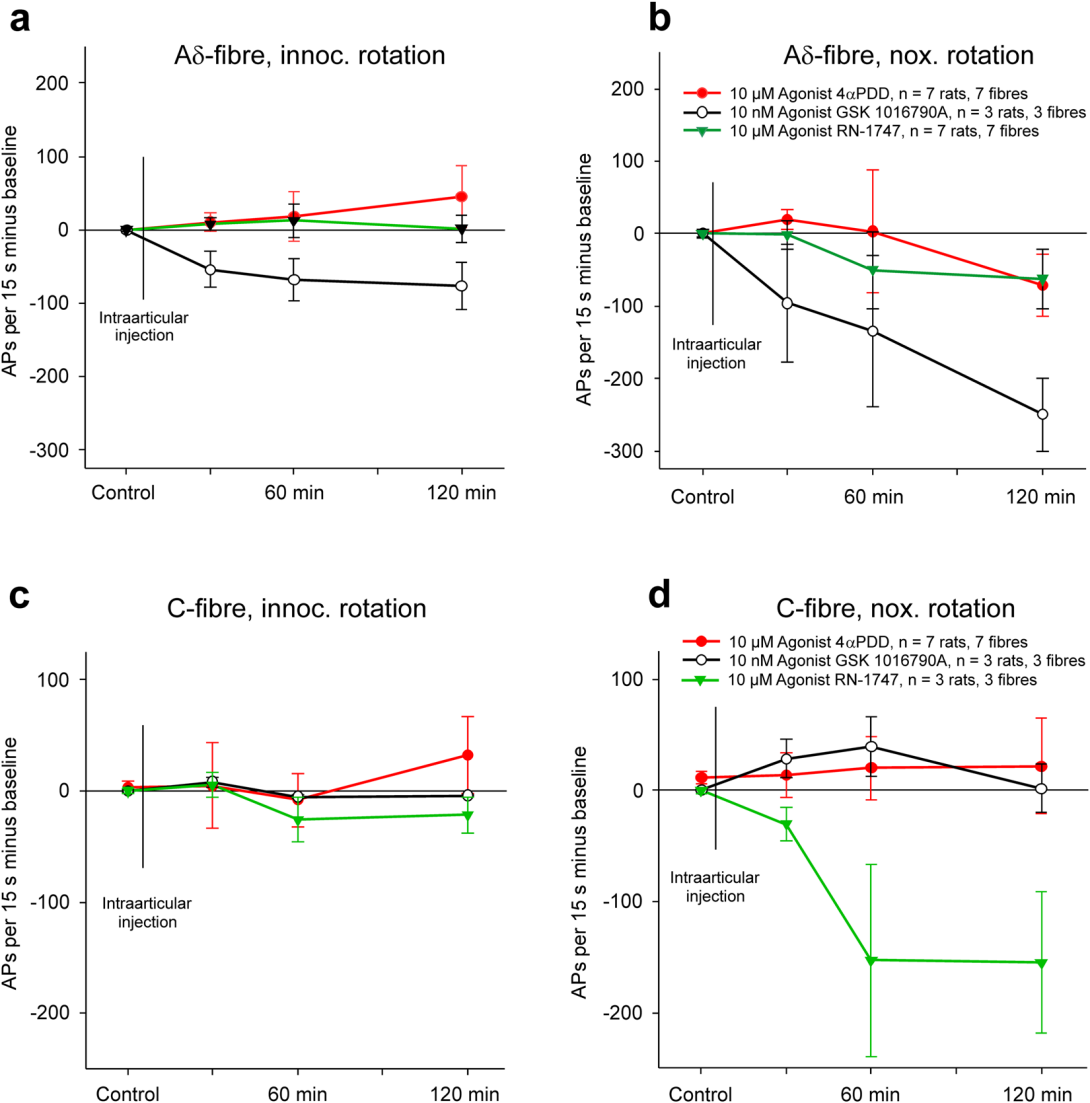
Influence of the TRPV4 agonists 4αPDD (10 µM), GSK 1016790 A (10 nM), or RN-1747 (10 µM) on the responses of A∂- and C-fibres to innocuous and noxious rotation of the normal knee joint. In all graphs the changes of the responses after injection of the agonists are shown, the baseline responses before the injections (control) were set zero. (**a**) Time course of the change of the responses of A∂-fibres to innocuous rotation after intraarticular injection of 4αPDD, GSK 1016790A, or RN-1747. Baseline values: 4αPDD group 65 ± 26 APs/15 s, GSK 1016790 A group 78 ± 32 APs/15 s, RN-1747 group 44 ± 15 APs/15 s. (**b**) Time course of the change of the responses of A∂-fibres to noxious rotation after intraarticular injection of 4αPDD, GSK 1016790A, or RN-1747. Baseline values: 4αPDD group 303 ± 68 APs/15 s, GSK 1016790A group 287 ± 40 APs/15 s, RN-1747 group 194 ± 48 APs/15 s. (**c**) Time course of the change of the responses of C-fibres to innocuous rotation after intraarticular injection of 4αPDD, GSK 1016790A, or RN-1747. Baseline values: 4αPDD group 109 ± 56 APs/15 s, GSK 1016790A group 9 ± 2 APs/15 s, RN-1747 group 29 ± 23 APs/15 s. (**d**) Time course of the change of the responses of C-fibres to noxious rotation after intraarticular injection of 4αPDD, GSK 1016790A, or RN-1747. Baseline values: 4αPDD group 139 ± 61 APs/15 s, GSK 1016790A group 98 ± 37 APs/15 s, RN-1747 group 203 ± 103 APs/15 s.

The effects of the agonists on the responses of the A∂-fibres is shown in Fig. 6a,b. None of the three agonists significantly influenced the responses of the A∂-fibres to innocuous rotation (Fig. 6a). The responses to noxious rotation were not altered by 4αPDD and RN-1747, but after GSK 1016790 A the responses to noxious rotation were reduced (Fig. 6b). Since GSK 1016790 A did not mimic the effects of 4αPDD and RN-1747, we did not test more than 3 fibres with GSK 1016790 A. In none of the groups the torque threshold was significantly altered.

Figure 6c,d show the average changes of the responses of C-fibres to innocuous and noxious rotation of the normal knee joint after intraarticular injection of the agonists. None of the three agonists significantly influenced the responses to innocuous rotation (Fig. 6c). The agonists 4αPDD and GSK 1016790 A had no effect on the responses to noxious rotation but the agonist RN-1747 reduced the responses to noxious rotation (Fig. 6d). Since RN-1747 had a different effect compared to 4αPDD and GSK 1016790 A, and since RN-1747 has also effects on TRPM8^20^, we did not test more than 3 neurons (and therefore no statistical evaluation was performed). In none of the groups the torque threshold was significantly altered.

### Effects of the TRPV4 receptor agonist 4αPDD on cultured DRG neurons

**Figure 7:**
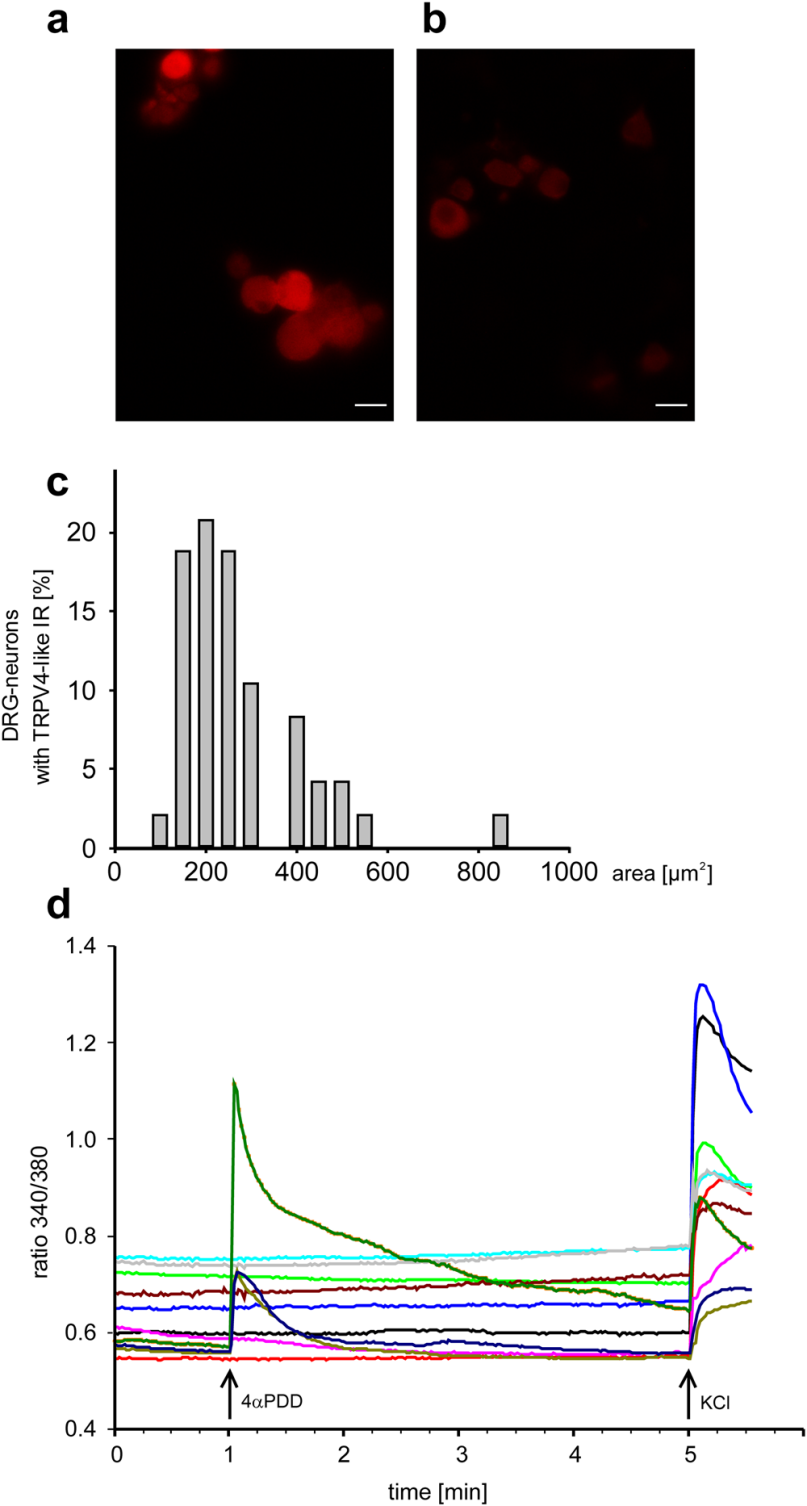
Labelling of cultured DRG neurons for TRPV4 and effect of 4αPDD on [Ca^2+^]_i_ of isolated and cultured DRG neurons. (**a**) Fixed DRG neurons labelled for TRPV4 with an anti-TRPV4 antibody. (**b**) Control experiment: No labelling of DRG neurons after adding control antigen to the primary antibody. (**c**) Size distribution of cultured DRG neurons expressing TRPV4. (**d**) Experiment in which 4αPDD induced a transient rise of [Ca^2+^]_i_ in 3 of 12 individual DRG neurons. All neurons showed also a typical potassium- (K^+^-) induced rise of [Ca^2+^]_i_ at the end of the experiment.

In addition to the recordings *in vivo*, we performed some experiments on cultured DRG neurons. First we explored on fixed DRG neurons the expression of TRPV4 using immunohistochemistry. Second, we explored the effect of 4αPDD on the intracellular [Ca^2+^] using calcium imaging. Figure 7a shows the labelling of TRPV4 in a proportion of DRG neurons with an anti-TRPV4 antibody whereas no labelled DRG neurons were seen after adding the control antigen to the primary antibody (Fig. 7b). The areas of the cell bodies of TRPV4-positive neurons are shown in Fig. 7c. The majority of the TRPV4-positive DRG neurons had areas in the range of 100–300 µm^2^ thus belonging to DRG neurons with C-fibres (n = 4 independent cultures).

In 5 independent cultures we found that 21 of 144 analysed DRG neurons showed a clear increase of [Ca^2+^]_i_. Figure 6a shows an experiment in which 4αPDDinduced a transient rise of [Ca^2+^]_i_ in 3 of 12 individual DRG neurons. All neurons displayed also the typical potassium- (K^+^-) induced rise of [Ca^2+^]_i_ (showing that cells were responsive) whereas the addition of buffer had no effect.

## Discussion

This study investigated the putative role of TRPV4 ion channels in joint pain and provided the following salient results. The intraarticular injection of a TRPV4 antagonist into the knee joint reduced the responses of C-fibres to noxious mechanical stimulation of the normal joint and the responses of sensitized C-fibres to innocuous and noxious mechanical stimulation of the acutely inflamed joint. The responses of A∂-fibres were not significantly altered by the antagonist, neither in the normal nor in the inflamed joint. The intraarticular application of TRPV4 agonists did not consistently alter the responses of A∂- and C-fibres to mechanical stimulation. Although the TRPV4 agonist 4αPDD induced a calcium influx in a proportion of isolated cultured DRG neurons, none of the agonists induced ongoing activity of the fibres *in vivo*. We conclude that TRPV4 ion channels are involved in the C-fibre-mediated mechanonociception of the normal joint and in the mechanical hypersensitivity of the inflamed joint.

In the normal joint the intraarticular application of the TRPV4 antagonist consistently reduced the responses of C-fibres to noxious (painful) rotation of the joint whereas the small responses of the C-fibres to innocuous rotation were not significantly reduced. The most likely interpretation is that noxious rotation opens TRPV4 channels and that the TRPV4 antagonist blocked the Ca^2+^ influx. During inflammation in the joint, C-fibres are sensitized and show stronger responses to innocuous rotation^1^. In the sensitized state also the increased responses to innocuous rotation were blocked suggesting that TRPV4 are involved in both the high threshold mechanonociception in the normal joint as well as in the sensitized mechanonociception in the inflammatory hyperalgesic state.

Interestingly, the TRPV4 antagonist did not alter the responses of A∂-fibres of the normal joint. Since we measured the conduction velocity of all recorded neurons we conclude that TRPV4 are predominantly expressed in C-fibres of the joint. However, since in the inflamed joint 2 of 7 fibres with conduction velocities of about 6 m/s showed a reduction of the responses after the antagonist, there might be some plasticity in the expression of TRPV4 in A∂-fibres (and in C-fibres). In synoviocytes of the rat temporomandibular joint and in the trigeminal ganglia TRPV4 was upregulated after intraarticular carrageenan injection^21^. We found that interleukin-17 which sensitizes C-fibres of the joint to mechanical stimuli and which affects mechanical but not thermal hyperalgesia^22–24^ up-regulated the expression of TRPV4 but not the expression of TRPV1 ion channels in cultured DRG neurons^22^.

Notably, however, in the present study the average responses of A∂-fibres to noxious movements did not decrease after RN-1734. The labelling of isolated DRG neurons showed that mainly small-sized neurons showed an expression of TRPV4. Since only few DRG neurons with an area >300 µm^2^ were labelled and since medium-sized neurons may be somata of either C- or A∂-fibres^25^, we did not make an effort to label DRG neurons supplying the joint. In the literature there are very few reports on the conduction velocities of sensory neurons expressing TRPV4. In electrophysiological recordings from primary bladder afferents only a proportion of C-fibres but not A∂-fibres were influenced by the TRPV4 agonist GSK1016790A and the TRPV4 antagonist RN-1734^26^. The lack of responsiveness of A∂-fibres to the antagonist clearly indicates that other mechanosensitive channels must also be involved in mechanonociception. Since both A∂- and C-fibres transmit noxious information to the spinal cord^1^, the application of an antagonist at TRPV4 channels will probably not have the ability to completely abolish joint pain evoked by mechanical stimuli.

Most of the data with the antagonist RN-1734 were obtained by using a dose of 500 µM. We chose this dose from the work of Wei *et al*.^27^ which was to our knowledge the only *in-vivo* application of this compound in studies on painful behaviour. Wei *et al*.^27^ injected RN-1734 into the skin of the face or into the paw of rats or applied it topically onto the dura mater at 500 µM. With this dose they found that the induction of allodynia by 4αPDD and RN-1747 could be prevented. Since we applied the compounds into the joint space we chose the same dose, because the joint is well perfused and allows a rapid exchange of substances from the joint. The lack of an effect of the antagonist at 500 µM on A∂-fibres indicates that TRPV4 ion channels are not involved in their responses to mechanical stimuli. However, since we found a reduction of the responses of C-fibres at 500 µM we explored whether a much lower dose, more close to the IC50 at 3.2 µM^28^ would also reduce the responses. As shown in Fig. 2e, the low dose of 20 µM RN-1734 reduced the responses of C-fibres of the normal joint to noxious rotation by the same magnitude as the high dose.

Because TRPV4 can also be activated chemically by specific agonists^19,28^ we explored whether the intraarticular application of TRPV4 agonists induces any noticeable activity or an effect on the responses to mechanical stimulation *in vivo*. The injection of the compounds did not elicit ongoing discharges nor did we consistently find an influence of these agonists on the magnitude of the responses to innocuous and noxious stimulation (exceptions see below). Since we used the responses to mechanical stimulation as readout parameter we were presumably not able to see an effect of the TRPV4 agonists because mechanical stimulation had opened the TRPV4 channel per se. On the other hand, these data raise doubts whether the chemical opening of the channel per se (as seen in the calcium imaging experiments) is sufficient to elicit action potentials. So far it is not precisely known how mechanical stimuli open TRPV4 ion channels. A complication of using agonists may be their complex effects. For example GSK1016790A regulates the membrane expression of TRPV4. In HEK293 cells GSK1016790A caused a calcium-dependent translocation and internalization of TRPV4 channels within 20 min^29^. Whether other agonists have similar effects is unknown. As mentioned above, RN-1747 may inhibit TRPM8 at an IC50 of 4 µM^20^.

Since DRG neurons express the TRPV4 channel^7,22,30^, and since specific TRPV4 agonists can evoke an increase of the intracellular Ca^2+^ concentration in isolated cultured DRG neurons (see also Segond von Banchet *et al*.^22^), it is likely that the TRPV4 antagonist blocked TRPV4 channels in the nerve endings in the joint. TRP channels including TRPV4 are transported from the cell body to the endings of the neurons^7^. However, TRPV4 channels are also expressed in other cells of the joint such as chondrocytes^31–33^ and synovial cells^34–39^. In chondrocytes, mechano/osmosensitive TRPV4 are involved in the sensing of osmolarity, and TRPV4 promotes cartilage extracellular matrix biosynthesis, upregulation of proanabolic and anticatabolic genes and increases in matrix accumulation^31–33^, and seems to be important for the maintenance of homeostasis in the cartilage. We cannot exclude, therefore, that the antagonist might have also influenced other cells than neurons in the joint. This would not change our conclusion that TRPV4 ion channels are involved in the C-fibre mediated mechanonociception because for the processing of information in the central nociceptive system the sensory outflow from the joint is important.

Antinociceptive effects of the TRPV4 antagonist which are involving other cells than neurons would imply that such cells release, upon activation of TRPV4 channels, mediators which then influence the neurons. This could be well the case because the effects of the antagonist were not immediately fully expressed after injection of the antagonist. This possibility should be further addressed if TRPV4 channels are considered as targets for treatment. In fact, TRPV4 channels in chondrocytes were discussed as targets for treatment, e.g. to prevent the development or progression of OA^31^ because insufficient or false functioning of TRPV4 in chondrocytes is thought to promote arthropathies^40,41^. However, to which extent TRPV4 protects against progression of osteoarthritis remains unclear^42,43^.

In summary, the present study provides evidence that TRPV4 ion channels significantly contribute to the responses of C-fibres to noxious mechanical stimulation of the normal joint, and to the responses of C-fibres to innocuous and noxious mechanical stimulation of the acutely inflamed joint. The responses of the majority of nociceptive A∂-fibres was not altered indicating that other mechanosensitive ion channels must be involved in mechano(noci)ception. The failure of the agonists to induce consistent changes of the responses to mechanical stimulation indicates that chemical stimulation of the TRPV4 channels is not sufficient to sensitize TRPV4 channels. More likely the sensitization of TRPV4 to mechanical stimuli is due to inflammatory mediators (see above).

## Material and Methods

### Electrophysiological recordings from joint afferents

This study was approved by the Thuringian Government (Thüringer Landesamt für Verbraucherschutz) and performed according to the Protection of Animals Act of the Federal Republic of Germany. The rats were treated in accordance with the declaration of Helsinki and the guiding principles in the care and use of animals. Data sampling, evaluation, and presentation complied with the ARRIVE guidelines.

Adult male Wistar rats (n = 73, age > 90 days, body weight 300–400 grams, supplied by the Animal Facility of the University Hospital Jena) were anesthetized with 100 mg/kg sodium thiopentone (Altana, Konstanz, Germany) i.p. and supplemental doses (20 mg/kg i.p.) were applied as necessary to maintain areflexia. Rats breathed spontaneously, mean arterial blood pressure, the electrocardiogram, and body temperature were controlled. The right thigh was exposed from knee joint to groin, a fastener fixed the right femur and the right hind paw was fixed in a shoe-like holder that allowed calibrated rotation of the lower leg in the knee joint. For testing responsiveness of fibres to mechanical stimulation we used innocuous outward torque (20 mNm, 15 s each) and noxious outward torque (40 mNm, 15 s each). The torque was applied by hand and was adjusted using the readout of a torque gauge (MVD2510, HBM Hottinger-Baldwin, Darmstadt, Germany).

Using platinum wire electrodes, action potentials were recorded from single fibres of the medial articular nerve (MAN) which had receptive fields in the knee joint. The local mechanical threshold in the receptive field was determined with calibrated von Frey hairs (1.6–52 g). The conduction velocity of the nerve fibres (C: ≤1.25 m/s, A∂: 1.25–10 m/s, Aβ: ≥10 m/s) was determined by electrical stimulation of the mechanical receptive field. Stimulation was performed with a bipolar electrode (pulses of 1–10 V and 0.5 ms duration) and the distance between electrode and recording site was measured. The recorded action potential signals were fed into a PC (interface card DAB 1200, Microstar Laboratories Inc., Bellevue, WA) that allowed the on-line construction of peristimulus time histograms and off-line analysis of action potentials using the spike/spidi software package^44^.

The recordings were grouped in blocks consisting of 1 min recording of spontaneous neuronal activity without any mechanical stimulation, followed by three applications of innocuous outward torque at the beginning of each following minute and three applications of noxious outward torque in the same time intervals (see specimen in Fig. 1d). The recording blocks started regularly at intervals of 15 min. After establishing the baseline responses (usually the first 4 test blocks) 0.1 mL of the test substance were injected into the knee joint cavity, and testing was continued in the same time schedule for three hours.

In 14 rats, an acute inflammation was induced in the right knee joint by injection of a kaolin suspension (Sigma-Aldrich, Taufkirchen, Germany; 4%, 0.1 mL) followed 15 min later by a carrageenan solution (Sigma; 2%, 0.1 mL) into the joint cavity. After 7–11 hours the same recording protocol was performed as described above. After recordings the rats were killed with 1.0 mL sodium thiopentone intravenously.

Test substances were the TRPV4 receptor agonist RN-1747 (Sigma-Aldrich), dissolved in dimethylsulphoxide (DMSO) and ethanol (EtOH) 6.3 mM as a stock solution and further diluted in isotonic NaCl, applied at 20 µM or 10 µM; the TRPV4 receptor agonist 4α-phorbol 12,13-didecanoate (4αPDD, Sigma-Aldrich), dissolved in PBS-buffer and EtOH to 100 µM as a stock solution, further diluted in PBS-buffer and applied at 10 µM; the TRPV4 receptor agonist GSK 1016790 A (Glaxo Smith Kline), dissolved in DMSO to 10 mM as a stock solution and further diluted in PBS-buffer, applied at 10 nM, and the TRPV4 receptor antagonist RN-1734 (Sigma-Aldrich), dissolved in isotonic NaCl and applied at 500 µM. The dose of the antagonist was taken from data of Wei *et al*.^27^. In addition, we tested a lower dose of only 20 µM of the antagonist RN-1734 in a further group of rats. Only one concentration of a substance was tested in each animal. The compound 4αPDD is a selective TRPV4 agonist which interacts directly with the ion channel, the EC50 in the rat is 4.4 µM^19^. RN-1747 has a similar efficacy and an EC50 of 4.1 µM in the rat, may however antagonize TRPM8 at an IC50 of 4 µM^19^. GSK 1016790 A has an EC50 of 0.01 µM^19^. The antagonist RN-1734 fully antagonizes effects of 4αPDD and RN-1747 and is selective for TRPV4 at an IC50 3.2 µM^19^.

### Primary culture of dorsal root ganglion neurons

Adult Wistar rats were sacrificed with a lethal dose of CO_2_. DRGs from all segments of the spinal cord were dissected. Ganglia were incubated at 37 °C with 215 U/mL collagenase type ΙΙ (Paesel & Lorei, Hanau, Germany) dissolved in Ham’s F12 medium (Gibco, BRL, Eggenstein-Leopoldshafen, Germany) for 100 min. After washing with Ca^2+^- and Mg^2+^- free PBS the ganglia were placed in D-MEM (Dulbecco’s modified Eagles medium; Gibco) containing 10 000 U/mL trypsin (Sigma), for 11 minutes at 37 °C. The cells were dispersed by gentle agitation and aspiration with a fire polished Pasteur-pipette. The dispersed cells were collected by centrifugation (500 × g, 8 min), washed 3 times in D-MEM and centrifuged again. The cell pellets were suspended in Ham’s F-12 medium containing 10% heat-inactivated horse serum (Gibco), 100 U/mL penicillin (Gibco), 100 µg/mL streptomycin (Gibco), and 10 ng/mL nerve growth factor (NGF, Paesel & Lorei), were collected by centrifugation and were plated on poly-L-lysine- (200 µg/mL) coated glass cover slips (diameter 13 mm). The cells were incubated at 37 °C in a humidified incubator gassed with 5.0% CO_2_ and air. The cells were fed after an overnight setting with standard medium (2 mL) and used in the next 24 hours.

### Labelling of cultured DRG neurons with an anti-TRPV4 antibody

The cover slips were transferred into 2% paraformaldehyde in 0.1 M/L phosphate buffer (pH 7.2) for 30 min. After washing with phosphate buffered saline (PBS) cells were autoclaved for 15 min (120 °C, 1 bar) in 0.1 M citrate buffer (pH 6.0). After cooling down and washing with PBS cells were incubated with 2% normal goat serum (Rockland, Gilbertsville, USA) in PBS TX-100. Thereafter the cells were incubated overnight with an anti-TRPV4 antibody (Alomone Labs, Jerusalem, Israel, # ACC-034) diluted 1:100 in PBS, plus 1% Triton-X100 and 1% gelatine from cold water fish skin at 4 °C in a moist chamber. After 3 times washing with PBS, cover slips were incubated for 2 hours at 20 °C with a Alexa-Fluor 568 goat-anti-rabbit antibody (Thermo Fisher Scientific Waltham, Massachusetts, USA, #A11011), diluted 1:200 in PBS, plus 1% Triton-X100 and 1% gelatine from cold water fish skin. After 3 times washing with PBS cells were dehydrated and embedded in Entellan. To test the specificity of the labelling, the control antigen (the peptide corresponding to amino acid residues 853–871 of rat TRPV4 against which the antibody was raised) was added to the primary antibody in a 10 fold excess, and after preincubation of the control antigen no labelling of DRG neurons was observed. Embedded DRG cells were analysed using a light microscope (Axioplan 2, Zeiss, Jena, Germany) coupled to an image analysing system (Axiovision, Zeiss). From each neuron the mean fluorescence value and the area were determined. All neurons were considered as positive if they showed a fluorescence value above that of neurons from the control incubation (see above). In total about 400 neurons were analysed (from n = 4 independent cultures).

### Ca^2+^-imaging

Cultured rat DRG neurons were loaded with 5 µM Fura-2 acetoxymethylester (Fura-2/AM, Molecular Probes, Leiden, The Netherlands), dissolved in dimethyl sulfoxide (DMSO) and 0.02% pluronic-127 detergent (Molecular Probes) which remained on the cover slips for 30 min at 20 °C. After incubation the neurons were washed several times with N-[2-hydroxyethyl]piperazine-N′-[2-ethanesulfonic acid] (HEPES) buffer [150 mM NaCl, 5 mM KCl, 10 mM glucose, 10 mM HEPES, 2 mM CaCl_2_, 2 mM MgCl_2_/(6H_2_O)] and then left in the buffer solution for about 20 min to complete cytoplasmatic dye esterification. The glass cover slips with dye-loaded cells were mounted on the stage of a fluorescence microscope (Zeiss) and superfused with HEPES buffer. The cells were illuminated alternately with light of 340 nm (specific for Fura-2 that has bound Ca^2+^) and 380 nm (specific for Ca^2+^-free Fura-2), and light of 510 nm was collected via a cooled CCD camera. The photomultiplier was coupled to a personal computer for data acquisition. Data were collected every 260 ms and stored in sequential files. The images were analysed using software from Zeiss (Axiovision). In each experiment, 340 nm/380 nm ratio values were calculated. To monitor local changes of [Ca^2+^]_i_, specific regions of interest were chosen and [Ca^2+^]_i_ for these regions was averaged and plotted as a function of time. Each experiment was started by stopping the superfusion. After 20 s 100 µL of the buffer solution plus 0.05% ethanol were added to the bath to exclude mechanical activation of the neurons. After 1 min 100 µL of 4αPDD (10 µM/L in HEPES buffer plus 0.05% ethanol, Sigma) was applied to the bath. To check the vitality of the neurons the cells were finally exposed to 50 mM KCl in HEPES buffer.

### Data evaluation and statistics

The action potentials were sorted by shape and amplitude. In the electrophysiological experiments *in vivo*, we counted the numbers of action potentials that occurred spontaneously in the one-minute-period before stimulation started and those that were evoked by mechanical stimulation. In some figures raw data are presented. In order to allow a comparison between different animals, we calculated the net changes in numbers of action potentials by normalizing to the baseline before application of a test substance. To do so, responses to the same stimuli and ongoing activity of the 1 min recording intervals of each animal were averaged throughout the baseline period. These means were then subtracted from each corresponding response/period throughout the application of the test substance in the particular animal. Then the responses to the stimuli of the same type within one block were averaged. In the end, data of animals which underwent the same treatment were averaged. As an additional parameter the mechanical threshold for torque (torque that induced a stable discharge rate of action potentials) was assessed at the beginning of the experiment and at the end of the observation period after application of the compound.

Values are given as mean ± SEM. For statistical analysis of changes within groups we used the Wilcoxon signed rank test or a paired t-test (Student). Significance was accepted at p < 0.05. Significances were calculated with the Instat software package (Graph Pad InStat, San Diego, USA).

## Data Availability

The datasets generated during and/or analysed during the current study are available from the corresponding author on reasonable request.

## References

[1] Schaible H-G, Grubb BD (1993). Afferent and spinal mechanisms of joint pain. Pain.

[2] Schaible H-G (2009). Joint pain. Exp. Brain Res..

[3] Eitner A (2017). Pain sensation in human osteoarthritic knee joints is strongly enhanced by diabetes mellitus. Pain.

[4] Liedtke W (2000). Vanilloid receptor-related osmotically activated channel (VR-OAC), a candidate vertebrate osmoreceptor. Cell.

[5] Strotmann R, Harteneck C, Nunnenmacher K, Schultz G, Plant TD (2000). OTRPC4, a non-selective cation channel that confers sensitivity to extracellular osmolarity. Nat. Cell Biol..

[6] Liedtke W, Friedman JM (2003). Abnormal osmotic regulation in trpv4^−/−^‐mice. Proc. Natl. Acad. Sci. USA.

[7] Levine D, Alessandri-Haber N (2007). TRP channels: Targets for the relief of pain. Biochimica Biophysica Acta.

[8] Alessandri-Haber N, Joseph E, Dina OA, Liedtke W, Levine JD (2005). TRPV4 mediates pain-related behavior induced by mild hypertonic stimuli in the presence of inflammatory mediator. Pain.

[9] Alessandri-Haber N (2003). Hypotonicity induces TRPV4-mediated nociception in rat. Neuron.

[10] Alessandri-Haber N, Dina OA, Joseph EK, Reichling D, Levine JD (2006). A transient receptor potential vanilloid 4-dependent mechanism of hyperalgesia is engaged by concerted action of inflammatory mediators. J. Neurosci..

[11] Todaka H, Taniguchi J, Satoh JI, Mizuno A, Suzuki M (2004). Warm temperature-sensitive TRPV4 plays an essential role in thermal hyperalgesia. J. Biol. Chem..

[12] Lee H, Iida T, Mizuno A, Suzuki M, Caterina MJ (2005). Altered thermal selection behavior in mice lacking transient receptor potential vanilloid 4. J. Neurosci..

[13] Suzuki M, Mizuno A, Kodaira K, Imai M (2003). Impaired pressure sensation in mice lacking TRPV4. J. Biol. Chem..

[14] Grant AD (2007). Protease-activated receptor 2 sensitizes the transient receptor potential vanilloid 4 in channel to cause mechanical hyperalgesia. J. Physiol..

[15] Hinata M (2018). Sensitization of transient receptor potential vanilloid 4 and increasing its endogenous ligand 5,6-epoxyeicosatrienoic acid in rats with monoiodoacetate-induced osteoarthritis. Pain.

[16] McDougall JJ, Hanesch U, Pawlak M, Schmidt RF (2001). Participation of NK1 receptors in nociceptin-induced modulation of rat knee joint mechanosensitivity. Exp. Brain Res..

[17] Gomis A (2013). Blockade of nociceptive sensory afferent activity of the rat knee joint by the bradykinin B2 receptor antagonist fasitibant. Osteoarthritis Cartilage.

[18] Schaible H-G (2014). Nociceptive neurons detect cytokines in arthritis. Arthritis Res. Ther..

[19] Vincent F, Dunction MAJ (2011). TRPV4 agonists and antagonists. Curr. Topic Med. Chem..

[20] Jankowski Michael P., Rau Kristofer K., Koerber H. Richard (2017). Cutaneous TRPM8-expressing sensory afferents are a small population of neurons with unique firing properties. Physiological Reports.

[21] Denadai-Souza A (2012). Role of transient receptor potential vanilloid 4 in rat joint inflammation. Arthritis Rheumatol..

[22] Segond von Banchet G (2013). Neuronal IL-17 receptor upregulates TRPV4 but not TRPV1 receptors in DRG neurons and mediates mechanical but not thermal hyperalgesia. Mol. Cell. Neurosci..

[23] Ebbinghaus, M. *et al*. Interleukin-17A is involved in mechanical hyperalgesia but not in the severity of murine antigen-induced arthritis. *Sci*. *Rep*., 10.1038/s41598-017-10509-5 (2017). 10.1038/s41598-017-10509-5 PMC5583382 28871176

[24] Richter F (2012). Interleukin-17 sensitizes joint nociceptors for mechanical stimuli and contributes to arthritic pain through neuronal IL-17 receptors in rodents. Arthritis Rheumatol..

[25] Lee KH, Chung K, Chung JM, Coggeshall RE (1986). Correlation of cell body size, axon size, and signal conduction velocity for individually labelled dorsal root ganglion cells in the cat. J. Comp. Neurol..

[26] Aizawa N, Wyndaele J-J, Homma Y, Igawa Y (2012). Effects of TRPV4 cation channel activation of the primary bladder afferent activities of the rat. Neurourology Urodynamics.

[27] Wei X, Edelmayer RM, Yan J, Dussor G (2011). Activation of TRPV4 on dural afferents produces headache-related behavior in a preclinical rat model. Cephalalgia.

[28] Vincent F (2009). Identification and characterization of novel TRPV4 modulators. Biochem. Biophys. Res. Commun..

[29] Baratchi, S. *et al*. The TRPV4 Agonist GSK1016790A regulates the membrane expression of TRPV4 channels. *Front*. *Pharmacol*, 10.3389/fphar.2019.00006 (2019). 10.3389/fphar.2019.00006 PMC6351496 30728775

[30] Chen Y (2013). Temporomandibular joint pain: a critical role for Trpv4 in the trigeminal ganglion. Pain.

[31] McNulty AL, Leddy HA, Liedtke W, Guilak F (2015). TRPV4 as a therapeutic target for joint diseases. Naunyn-Schmiedeberg´s Arch. Pharmacol..

[32] Krupkova O, Zvick J, Wuertz-Kozak K (2017). The role of transient receptor potential channels in joint diseases. *Eur*. Cells Materials.

[33] O’Conor CJ, Leddy HA, Benefield HC, Liedtke B, Guilak F (2014). TRPV4-mediated mechanotransduction regulates the metabolic response of chondrocytes to dynamic loading. Proc. Natl. Acad. Sci. USA.

[34] Itoh Y (2009). An environmental sensor, TRPV4 is a novel regulator of intracellular Ca2+ in human synoviocytes. Am. J. Physiol. Cell Physiol..

[35] Kochukov MY, McNerney TA, Fu Y, Westlund KN (2006). Thermosensitive TRP ion channels mediate cytosolic calcium response in human synoviocytes. Am. J. Physiol. Cell Physiol..

[36] Kochukov Mikhail Y, McNearney Terry A, Yin Huaizhi, Zhang Liping, Ma Fei, Ponomareva Larissa, Abshire Sarah, Westlund Karin N (2009). Tumor Necrosis Factor-Alpha (TNF-α) Enhances Functional Thermal and Chemical Responses of TRP Cation Channels in Human Synoviocytes. Molecular Pain.

[37] Masuyama R (2008). TRPV4-mediated calcium influx regulates terminal differentiation of osteoclasts. Cell Metab..

[38] Mizoguchi F (2008). Transient receptor potential vanilloid 4 deficiency suppresses unloading-induced bone loss. J. Cell Physiol..

[39] Phan MN (2009). Functional characterization of TRPV4 as an osmotically sensitive ion channel in porcine articular chondrocytes. Arthritis Rheumatol..

[40] Nilius B, Voets T (2013). The puzzle of TRPV4 channelopathies. EMBO Rep..

[41] Lamandé SR (2011). Mutations in TRPV4 cause an inherited arthropathy of hands and feet. *Nat*. Genetics.

[42] Clark AL, Votta BJ, Kumar S, Liedtke W, Guilak F (2010). Chondroprotective role of the osmotically sensitive ion channel transient receptor potential vanilloid 4. Arthritis Rheumatol..

[43] O’Conor, C. J. *et al*. Cartilage-specific knockout of the mechanosensory ion channel TRPV4 decreases age-related osteoarthritis. *Sci*. *Rep*, 10.1038/srep29053 (2016). 10.1038/srep29053 PMC4937413 27388701

[44] Forster C, Handwerker HO (1990). Automatic classification and analysis of microneurographic spike data using a PC/AT. J. Neurosci. Methods.

